# Analysis of cell-free DNA in a consecutive series of 13,607 routine cases for the detection of fetal chromosomal aneuploidies in a single center in Germany

**DOI:** 10.1007/s00404-020-05856-0

**Published:** 2020-11-05

**Authors:** Heike Borth, Anna Teubert, Ralf Glaubitz, Sarah Knippenberg, Nargül Kutur, Thomas Winkler, Bernd Eiben

**Affiliations:** 1 Amedes Institut für Labormedizin und Klinische Genetik Rhein/Ruhr, Willy Brandt Platz 4, 45127 Essen, Germany; 2 Amedes Genetics, Georgstr. 50, 30159 Hannover, Germany

**Keywords:** Noninvasive prenatal testing, Fetal chromosomal aneuploidies, Fetal fraction, Positive predictive value, VeriSeq NIPT Solution

## Abstract

**Purpose:**

Noninvasive prenatal testing (NIPT) is a highly sensitive and specific method for detection of fetal chromosomal aneuploidies from maternal plasma. The objective of this study was to determine the performance of a new paired-end sequencing-based NIPT assay in 13,607 pregnancies from a single center in Germany.

**Methods:**

Samples from 13,607 pregnant women who previously underwent NIPT were analyzed using VeriSeq NIPT Solution v2 assay for presence of common fetal trisomies and monosomy X. Follow-up to determine clinical truth was carried out.

**Results:**

Of the 13,607 cases, 13,509 received a NIPT call resulting in a low study failure rate of 0.72%. There were 188 (1.4%) high-risk calls: 117 trisomy 21, 34 trisomy 18, 23 trisomy 13, one trisomy 21 + 13, and 13 monosomy X. High sensitivities and specificities of ≥ 98.89% were reported for all four aneuploidy conditions. Of the high-risk cases, clinical follow-up data were available for 77.1% (145/188). Clinical follow-up of high-risk calls revealed an overall positive predictive value of 84.8% (potential range 65.4–88.3%). NIPT results were provided for samples across a range of fetal fractions, down to 2% fetal fraction.

**Conclusion:**

The VeriSeq NIPT Solution v2 assay detected fetal chromosomal aneuploidies across a range of fetal fractions with high sensitivities and specificities observed based on known clinical outcomes, a high overall PPV, and a low failure rate.

**Electronic supplementary material:**

The online version of this article (10.1007/s00404-020-05856-0) contains supplementary material, which is available to authorized users.

## Introduction

The discovery of fetal cell-free DNA (cfDNA) in maternal circulation in 1997 [1] facilitated the development and commercial availability of noninvasive prenatal testing (NIPT) assays to screen for the presence of fetal chromosomal anomalies. These NIPT assays have been shown to have superior performance over traditional serum screening methods [2]; a recent meta-analysis found that NIPT could detect at least 98% of common fetal trisomies in singleton pregnancies with a combined false-positive rate of 0.13% [3]. The use of NIPT in the general pregnancy population has been endorsed by a number of professional societies, including the German Society of Human Genetics [4–7], and recommendations from German Society for Ultrasound in Medicine (DEGUM), Austrian Society for Ultrasound in Medicine (ÖGUM), Swiss Society for Ultrasound in Medicine (SGUM), and Fetal Medicine Foundation (FMF) Germany regarding prenatal screening including cfDNA screening were recently published [8]. The 2015 Austria–Swiss–German guidelines for NIPT endorse the use of NIPT to screen for fetal trisomy 21 in all pregnant women, but do not recommend the screening of sex chromosome aneuploidies or microdeletions without reservation [9]. The guidelines note that the performance of cfDNA screening for trisomy 13 and 18 is lower than that observed for trisomy 21. Germany’s publicly funded insurance system plans to cover NIPT for common fetal trisomies in pregnancies with increased need of surveillance and pregnancies with special risks in the near future.

The range of conditions that NIPT assays can screen for has expanded since their commercial availability in 2011. Initially, NIPT assays screened for the presence of common fetal trisomies only, i.e., trisomy 21, trisomy 18, and trisomy 13 [10, 11]. This was then expanded to include optional testing for fetal sex and sex chromosome aneuploidies [12, 13], and later to include the option of screening for common microdeletions [14, 15], rare autosomal aneuploidies, and partial deletions and duplications [16–18]. The additional fetal anomalies that are captured following genome-wide testing can impact patient management as they are often associated with serious pregnancy complications [16, 19]. The ability of commercially available NIPT assays to detect additional fetal anomalies is dependent on the technology used by the assay. The primary NIPT technologies at present include massively parallel whole-genome next-generation sequencing (NGS) [20–22], single-nucleotide polymorphism (SNP)-based targeted sequencing [23, 24], and microarray sequencing [25], of which only some NGS-based assays are currently validated to detect rare autosomal aneuploidies and partial deletions and duplications. Massively parallel sequencing techniques can use either single-end or paired-end sequencing; the use of paired-end sequencing is advantageous as it provides information on both the DNA fragment size and location. Several studies have shown the use of this type of sequencing in the noninvasive screening of fetal chromosomal aneuploidies [26, 27], and the first paper on clinical use of paired-end sequencing was published in 2017 [28].

The fetal fraction (FF) of a pregnancy plasma sample has been shown to play a key role in NIPT [29]. Patient characteristics can impact the fetal fraction level in NIPT samples; a positive correlation between gestational age and fetal fraction and a negative correlation between maternal weight and fetal fraction have been shown [24, 30, 31]. Low fetal fraction has been found to be one of the main reasons for NIPT assay failures. Studies have also shown that samples with very low fetal fraction have an increased risk of aneuploidy, particularly trisomy 18 and trisomy 13 [23, 32–34]; this is important as some NIPT assays apply a fetal fraction cut-off and, thus, may miss a number of affected pregnancies.

Our laboratory previously reported on the performance of a SNP-based NIPT assay in the analysis of cfDNA in 3000 cases [35]. The objective of the current study was to determine the performance of the VeriSeq™ NIPT Solution v2 assay, a paired-end sequencing-based NGS assay, in testing for fetal chromosomal aneuploidies in 13,607 general pregnancy samples. We also wanted to investigate the role of fetal fraction in our study, and to determine if patient characteristics had an impact on study outcomes.

## Materials and methods

### Study patient/sample details

This retrospective analysis study involved 13,607 consecutive patients undergoing NIPT from the general pregnancy population collected over a 17-month period between December 2017 and April 2019. Both singleton and twin pregnancy samples of at least 10 weeks’ gestation were included in the study; exclusion criteria included a known vanishing twin or a higher-grade multiple pregnancy. For this study, the existing sequencing files from the original sample analysis were reanalyzed using the bioinformatic pipeline of the new NIPT assay, VeriSeq NIPT Solution v2, being implemented in the laboratory. Results from the reanalysis were not communicated to the patients or their physicians. Amedes observes the provisions of the Federal Data Protection Act. Patient consent was obtained from all patients involved in the study for use of their data for appropriate quality control and improvements for NIPT assays. In addition, all data were deidentified before being included in the study.

Indications for NIPT included advanced maternal age, a positive screening test result (ultrasound, serum marker), other medical reasons, or patient anxiety. Other medical reasons included an abnormal ultrasound, known disease such as diabetes, epilepsy, and carcinoma, medication such as chemotherapy, a previous history of pregnancy complications including miscarriage or a previously affected pregnancy (e.g., trisomy 21, 18, 13, monosomy X), a genetic aberration in the family (e.g., trisomy 21), or consanguinity. If no information about the indication was provided, patient anxiety or advanced maternal age (if the patent was ≥ 35 years) was taken as the indication. If several of the above mentioned indications were provided, the cases were assigned to the indication groups with the following priority to assign only one indication group to each patient: (1) positive screening test result, (2) advanced maternal age, (3) other medical reasons, and (4) patient anxiety. The correctness of the classification of the cases to the different indication groups, as well as all other information of the patient anamnesis and the feedback regarding the clinical outcome, was dependent on the correctness of the information received.

Clinical outcomes, i.e., clinical truth, for study cases were determined by invasive prenatal diagnostic techniques (cytogenetic analysis after chorionic villus sampling (CVS) and/or amniocentesis), products of conception analysis (cytogenetic analysis of the abortion tissue or placenta samples, post-mortem examination such as autopsy or macroscopic assessment of the abortion), and postnatal cytogenetic analysis, as well as by ultrasound and newborn physical exam. NIPT results positive for fetal aneuploidy were considered confirmed when validated by either invasive prenatal diagnostics or an anomaly observed on ultrasound that matched the high-risk NIPT call. Low-risk NIPT results were considered confirmed if the attending physician provided feedback on a healthy born child with no or minor anomalies that did not clearly fit trisomy 21, 18, 13 or monosomy X (e.g., clubfeet, renal pelvic dilatation, heart defects such as ventricular septal defect, or intrauterine growth restriction due to placental insufficiency).

### VeriSeq NIPT Solution v2 assay

NIPT analysis was carried out using the VeriSeq NIPT Solution v2 bioinformatic pipeline (Illumina Inc., San Diego, CA, USA). This assay uses a paired-end sequencing technique and has two reporting modes: Basic, with reporting for common trisomies and sex chromosomes (if selected), and Genome-wide analysis for detection of the presence of genome-wide fetal anomalies (including rare autosomal aneuploidies and partial deletions and duplications ≥ 7 Mb). As study patients originally consented to the analysis of their samples for common trisomies and sex chromosomes (if selected), results for anomalies other than those screened for using basic mode are not reported in this study. Following analysis of existing sequencing files by the VeriSeq NIPT Assay Software v2, samples were called as Anomaly Detected or No Anomaly Detected for common fetal trisomies (trisomy 21, trisomy 18, and trisomy 13) as well as monosomy X (singletons only). A fetal fraction estimate of the sample in question was also provided.

Fetal fraction was estimated using observed coverage data and fragment size distribution. The coverage-based estimate was obtained using methodology similar to a previously described method [36]. Size-based FF estimates were based on the fact that fetal cfDNA fragments are, on average, shorter than maternal fragments [27, 36, 37].

Following data analysis, a log likelihood ratio (LLR) score was provided for each sample. This is the probability of a sample being affected given the sample’s estimated FF and observed coverage. The assay software also uses a dynamic threshold metric known as the individualized Fetal Aneuploidy Confidence Test (iFACT), which determines whether there is sufficient sequencing coverage for each individual sample given the FF estimate for that sample; samples that did not meet this threshold were reported as QC failures. Other QC metrics such as uniformity of coverage were also taken into account. The assay also provided a T-Statistics value, which was used to help differentiate between low-risk and high-risk samples.

### Statistics

Statistical data analysis was performed using Microsoft Excel 2010. Where applicable, statistical significance of differences was assessed by Student’s *t* test; a *p* value < 0.05 was considered to be significant. Binomial 95% confidence intervals (CI) were calculated for sensitivity and specificity estimates.

In case of an inconclusive NIPT result of the first blood sample a second blood sample was taken for re-analysis. In these cases, the result of the first analysis was excluded in the statistical analysis, so that only one result per patient was included in the statistical evaluation. In addition, in some patients where a NIPT result could be obtained in the analysis of a first blood sample, but a low FF of less than 4% (3% FF or 2% FF) was present, another blood sample was taken for repeat analysis, which was processed as a gesture of goodwill. Again, in these cases, the result of the first analysis was excluded from the statistical analysis, with the exception of cases in which the repeat analysis did not yield a NIPT result; in the latter cases, the result of the first analysis was included in the statistics with a FF of 2% or 3%, and the unsuccessful repeat analysis was excluded.

## Results

**Fig. 1:**
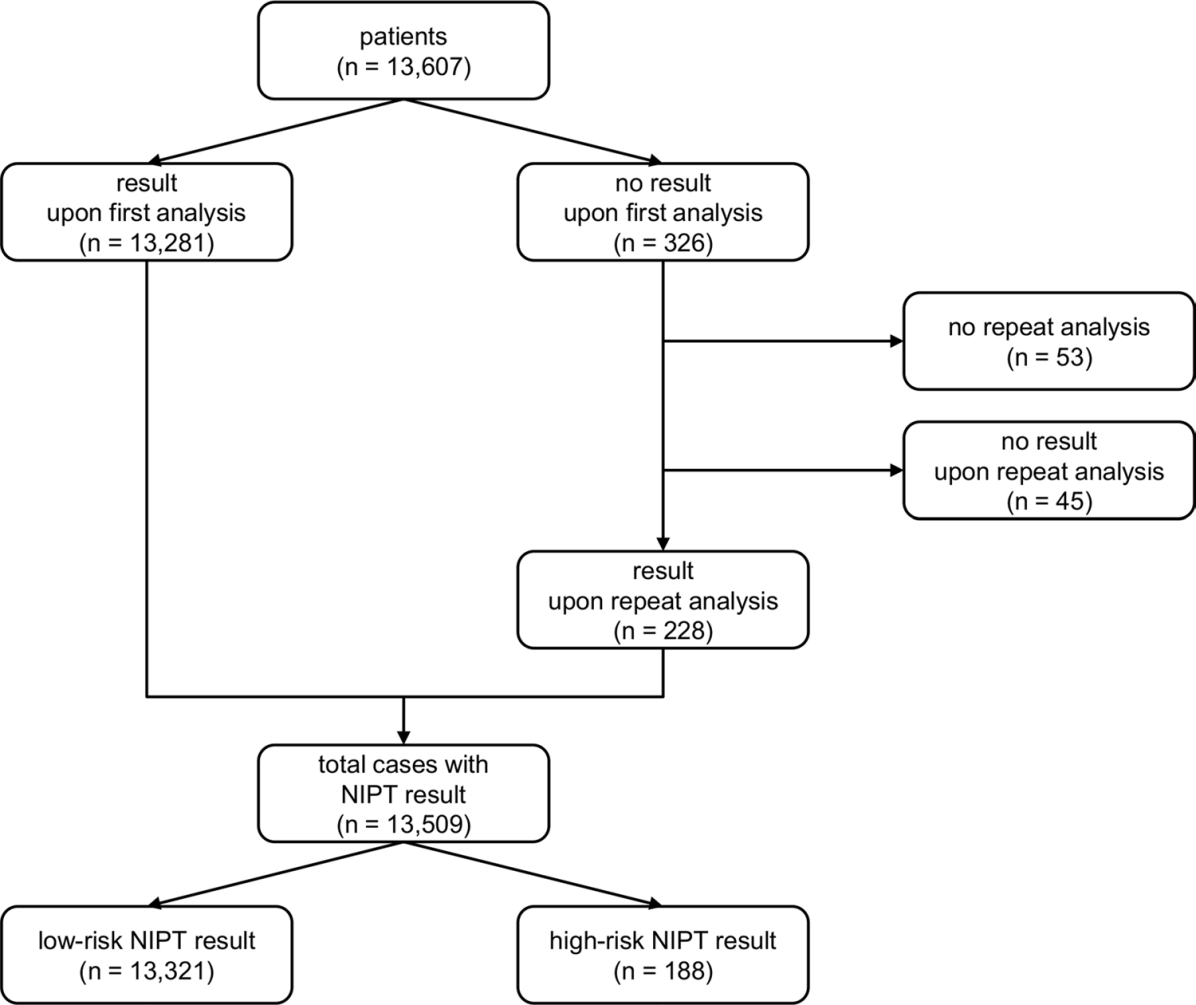
Flowchart of study samples

A total of 13,607 pregnancy cases were included in the study, which included 13,333 singleton and 274 twin pregnancy samples. Of these, 13,281 (97.6%) got an NIPT result upon analysis of the first blood sample. Of the 326 samples that did not get a result using the initial blood sample, 273 underwent a repeat analysis on a second blood sample, with 228 (83.5%) cases getting a result. Overall, a total of 13,509 patients obtained an NIPT result (Fig. 1); the study failure rate was 0.72% (98/13,607). Clinical outcomes were available for 2623 cases; outcomes were based on invasive diagnostic techniques, ultrasound, or new-born physical exam (Supplementary Table 1; Online Resource 1).

**Fig. 2:**
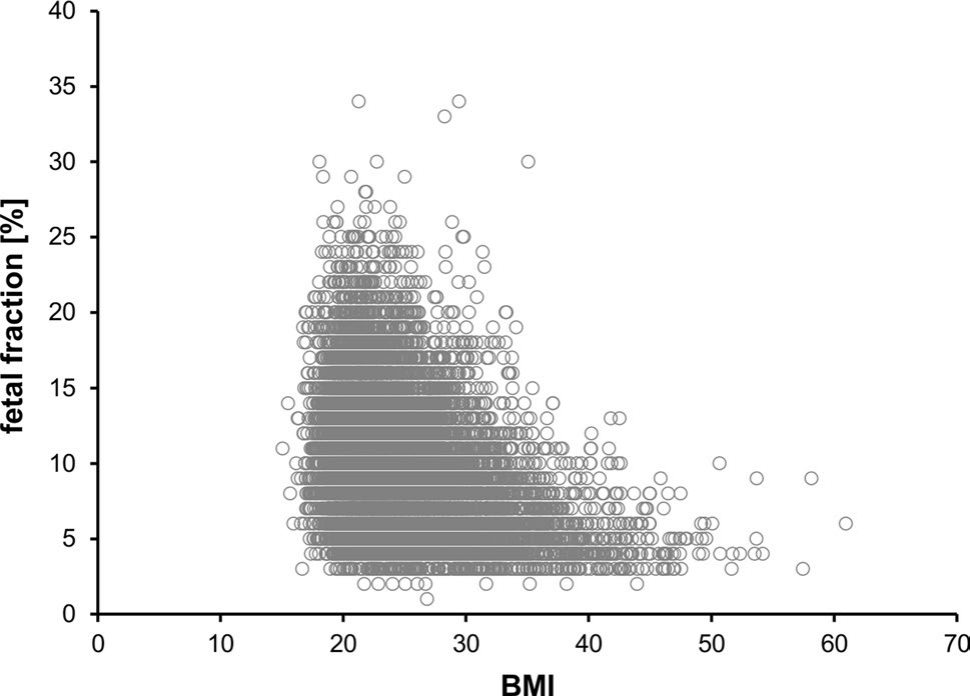
Relationship between fetal fraction and patient BMI

Patient demographics for the 13,607 patients included in the overall study are shown in Table 1. Almost half of all patients initially chose to undergo NIPT due to parental anxiety. The mean fetal fraction of the study samples was 9.62%, with a range of 1.20–33.94%. The relationship between fetal fraction and patient BMI is shown in Fig. 2. Overall, 86.6% of patients presented for testing in their first trimester; the relationship between gestational age and other patient characteristics (maternal age, BMI, and fetal fraction) is shown in Supplementary Fig. 1 (Online Resource 2). As expected, the BMI increased with increasing gestational age as did the fetal fraction; a marked increase of fetal fraction was observed only from > 20 weeks of gestation, whereas in the previous weeks of gestation a significant but only slight increase in the fetal fraction was observed. The relationships between indications for screening and maternal age, BMI, and fetal fraction were also investigated (Supplementary Fig. 2; Online Resource 2).

**Table 1:** Patient demographics for the study cohort

	Study cohort (*n* = 13,607)
Maternal age (year)	
Mean ± SEM	33.68 ± 0.04
Range	17.45–55.83
Gestational age (week)	
Mean ± SEM	12.48 ± 0.02
Range	10.00–36.57
BMI	
Mean ± SEM	24.87 ± 0.05
Range	15.05–60.96
Indication for screening [*n* (%)]	
Advanced maternal age	5755 (42.3)
Positive screening test result^a^	819 (6.0)
Other medical reasons^b^	748 (5.5)
Patient anxiety	6285 (46.2)

*Yr* year, *wk* week, *SEM* standard error of the mean, *BMI* body mass index

^a^Positive screening test result includes ultrasound or serum marker screening

^b^Other medical reasons include e.g., abnormal ultrasound, known diseases of the patient (e.g., diabetes, epilepsy, and carcinoma), medication (e.g., chemotherapy), or a high-risk family medical history such as a previous miscarriage, a genetic aberration in a previous pregnancy (e.g., trisomy 21, 18, 13, monosomy X), a genetic aberration in the family (e.g., trisomy 21), or consanguinity

Of the 13,509 patients that received a NIPT result, 98.6% (13,321) were called as low risk, and 1.4% (188) as high risk for presence of a fetal chromosomal aneuploidy (Fig. 1). Clinical outcomes were available for 2623 cases (Supplementary Table 1; Online Resource 1). Of the 13,321 cases reported as low-risk for fetal aneuploidy, clinical outcomes were available for 2478 cases (18.6%). Of these, there was one false-negative call that was found to be a trisomy 21 case; the fetal fraction of this false-negative case was 3%. Of the 188 high-risk calls, 117 were trisomy 21, 34 were trisomy 18, 23 were trisomy 13, one was a trisomy 21 + 13, and 13 were monosomy X calls; 1.3% of patients in this study received a positive NIPT call for presence of a classic trisomy. Follow-up was available for 77.1% (145/188) of these high-risk cases. Based on 123 true-positive and 22 false-positive cases, sensitivities of ≥ 98.89% and specificities of ≥ 99.73% were observed for the high-risk cases as shown in Table 2; results for the one trisomy 21 + 13 case are not shown but this case was found to be a true positive. An overall PPV of 84.8% (123/145) for high-risk cases was observed; PPV results for trisomy 21, trisomy 18, trisomy 13 and monosomy X cases are shown in Table 2. A theoretical PPV range was calculated based on the assumption that high-risk cases without follow-up were either all true positives or all true negatives; the theoretical PPV range for high-risk cases was 65.4% (123/188)–88.3% (166/188). Stratification of NIPT results by maternal age, BMI, and FF is shown in Supplementary Fig. 3 (Online Resource 2); trisomy 18 and trisomy 13 were found to be associated with a significantly lower fetal fraction (*p* < 0.01 and *p* < 0.001, respectively, compared to low-risk NIPT results). Fetal fractions of all low-risk and high-risk NIPT cases stratified by patient BMI are shown in Supplementary Fig. 4 (Online Resource 2). The LLR scores and T-Statistics values for the true-positive and false-positive high-risk cases are shown in Supplementary Table 2 (Online Resource 1). As can be seen, both the LLR score and the T-Statistics value were much lower for the false-positive trisomy 21 cases than for the true-positive trisomy 21 cases.

**Table 2:** Sensitivities, specificities, and positive predictive values for the high-risk NIPT cases

	Trisomy 21	Trisomy 18	Trisomy 13	Monosomy X
Cases (*n*)	117	34	23	13
Cases with follow-up [*n* (%)]	96 (82.1)	23 (67.6)	15 (65.2)	10 (76.9)
Sensitivity [% (*n*/*N*; 95% CI)]	98.89 (89/90; 93.96–99.97)	> 99.99 (19/19; 82.35–100)	> 99.99 (9/9; 66.37–100)	> 99.99 (5/5; 47.82–100)
Specificity [% (*n*/*N*; 95% CI)]	99.73 (2566/2573; 99.44–99.89)	99.84 (2496/2500; 99.59–99.96)	99.76 (2486/2492; 99.48–99.91)	99.80 (2482/2487; 99.53–99.93)
PPV [% (*n*/*N*)]	92.7 (89/96)	82.6 (19/23)	60.0 (9/15)	50.0 (5/10)
Theoretical lower PPV [% (*n*/*N*)]	76.1 (89/117)	55.9 (19/34)	39.1 (9/23)	38.5 (5/13)
Theoretical upper PPV [% (*n*/*N*)]	94.0 (110/117)	88.2 (30/34)	73.9 (17/23)	61.5 (8/13)

**Fig. 3:**
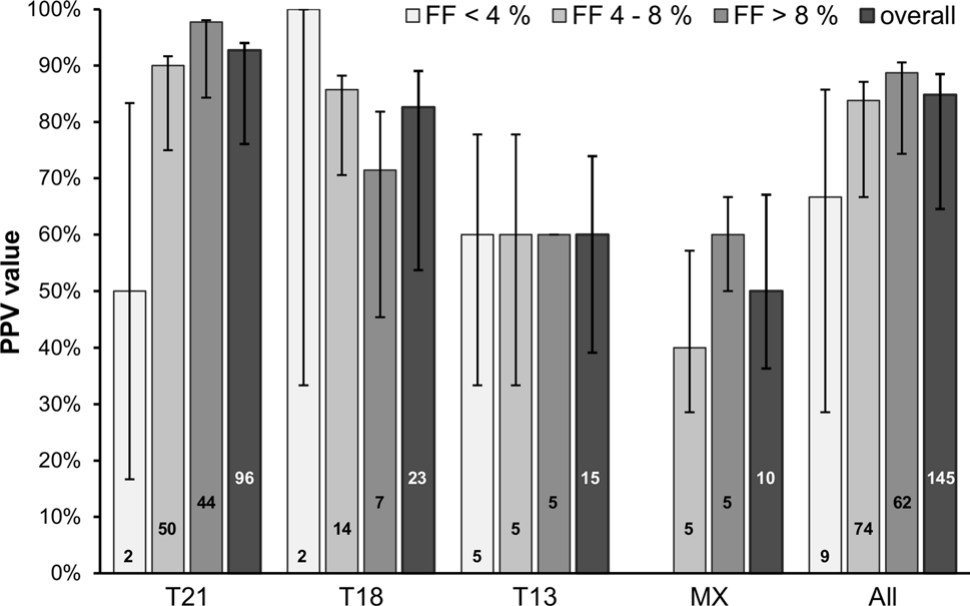
PPV values for high-risk cases for cases with fetal fractions < 4%, 4–8%, and > 8%

Overall, 2.0% (264/13,509) of samples had a fetal fraction less than 4%, 43.9% (5933/13,509) of samples had a fetal fraction between 4 and 8%, and 54.1% (7312/13,509) of samples had a fetal fraction greater than 8%. The breakdown of high-risk NIPT results by fetal fraction is shown in Fig. 3. As can be seen, the majority of high-risk cases had a fetal fraction between 4 and 8% (49.5%; 93/188). Of the 264 samples with a fetal fraction < 4%, 21 were classified as high-risk by NIPT—six trisomy 21, six trisomy 18, and nine trisomy 13. Outcomes were available for nine of these 21 cases resulting in a PPV of 66.7% (6/9). For low-risk cases, an NPV of 98.1% (52/53) for cases with a fetal fraction of less than 4% was observed. An NPV of > 99.9% was observed for cases with a fetal fraction between 4 and 8% (1198/1198), and for cases with a fetal fraction of ≥ 8% (1227/1227).

## Discussion

Here, we show that the VeriSeq NIPT Solution v2 assay was able to screen for fetal chromosomal aneuploidies with high sensitivities and specificities observed based on known clinical outcomes. The sensitivities and specificities reported in our study are in line with those found in a recent meta-analysis by Gil et al. [3] Our study also had a low overall failure rate of less than 1%. For clinical studies, the positive predictive value is a key metric that should be reported along with the assay sensitivity and specificity, provided that clinical follow-up is possible; here, clinical truth was available for the majority of high-risk cases. High PPVs were observed in our study, particularly for trisomy 21 and trisomy 18 cases, at levels similar to those shown in previous studies [22, 24, 38]. The limited follow-up of the low-risk cases in our study found one false-negative case that was determined to be a trisomy 21 case.

We also looked at the impact of patient characteristics on study outcomes. We observed a positive correlation between gestational age and fetal fraction, which has been noted in several previous studies [24, 30, 31]. As expected, a positive correlation was seen between gestational age and BMI. We also noted that there was a significantly lower fetal fraction in cases that screened positive for trisomy 18 and trisomy 13. As the cfDNA that is analyzed in NIPT assays is derived from the placenta, the smaller placental volume that has previously been reported in pregnancies affected with either trisomy 18 or trisomy 13 [39] is likely the cause of the lower FF levels observed in our study for these trisomic conditions.

One of the strengths of our study is that it involves a large cohort of patients from a general pregnancy population, providing further evidence for the utility of NIPT in that patient population. We demonstrate the high test performance of this NIPT assay, with high sensitivities, specificities, and PPVs reported for the NIPT-positive cases. We also report a low assay failure rate of less than 1% following retesting of a second blood sample for any samples that did not get a result with the initial blood sample. Having a low failure rate is an important feature for any NIPT assay as it allows patients to avoid having to decide whether or not to undergo an invasive diagnostic test without an NIPT result which can add to parental anxiety; professional societies often recommend that failed NIPT samples should be considered as high risk [4, 40, 41]. A limitation of our study was the inability to report on the additional findings of the genome-wide assay due to the retrospective nature of the study. This meant that we were unable to provide any performance data relating to the screening of rare autosomal aneuploidies and partial deletions and duplications. The ability of this NIPT assay to screen for fetal chromosomal aneuploidies even at very low fetal fractions is important, as cases with low fetal fraction have been shown to be associated with adverse outcomes [42]. However, the small number of samples in our study that had a fetal fraction less than 4% precluded any meaningful analysis of FF performance at these low levels. Another limitation of this study was that outcome information was limited for cases with a negative NIPT result, which is consistent with many other published NIPT studies [22, 24].

Currently, there is no insurance coverage for NIPT in Germany and so patients need to pay for NIPT out-of-pocket which can be prohibitive in many cases. However, Germany’s publicly funded health insurance system plans to cover NIPT for trisomies 21, 13, and 18 for particular pregnancies with increased need of surveillance and pregnancies with special risks. Introduction of nationwide health coverage for NIPT has been shown to result in a considerable increase in update of this type of prenatal screening for women with increased risk of fetal aneuploidy [43, 44]. It is important that patients are informed that NIPT is not a diagnostic test, and that high-risk NIPT calls should be confirmed by invasive diagnostic procedures such as CVS or amniocentesis. Patients who are found to be at high-risk following an abnormal ultrasound should be recommended to undergo an invasive diagnostic procedure to avoid any incorrect NIPT results that can occur due to reasons such as confined placental mosaicism [45].

In conclusion, the VeriSeq NIPT Solution v2 assay showed strong performance for the detection of fetal chromosomal aneuploidies in our large population of general pregnancy patients across a wide range of fetal fractions and can help to avoid unnecessary invasive procedures that risk harming pregnant woman.

## Electronic supplementary material

Below is the link to the electronic supplementary material.

Supplementary file1 (DOCX 56507 kb)

Supplementary file2 (PDF 109 kb)
