# Supplementary material for: Analysis of cell-free DNA in a consecutive series of 13,607 routine cases for the detection of fetal chromosomal aneuploidies in a single center in Germany
Source: Arch Gynecol Obstet. 2020 Nov 5;303(6):1407–14. doi: 10.1007/s00404-020-05856-0 (PMC8087552; doi:10.1007/s00404-020-05856-0)

**SUPPLEMENTARY FIGURES**

**Article Title:** Analysis of cell-free DNA in a consecutive series of 13,607 routine cases for the detection of fetal chromosomal aneuploidies in a single center in Germany

**Journal Name:** Archives of Gynecology and Obstetrics

**Author Names:** Heike Borth^1^, Anna Teubert^2^, Ralf Glaubitz^2^, Sarah Knippenberg^2^, Nargül Kutur^1^, Thomas Winkler^1^, Bernd Eiben^1*^

**Author Affiliations:**

^1^amedes Institut für Labormedizin und Klinische Genetik Rhein/Ruhr, Essen, Germany

^2^amedes genetics, Georgstr. 50, D-30159 Hannover, Germany

**Corresponding Author:***Bernd Eiben, [eiben@eurogen.de](mailto:eiben@eurogen.de)

**Supplementary Fig. 1**. Relationship between gestational age and maternal age (A), body mass index (B), and fetal fraction (C). Scatter plot (left) and mean values ± SEM (right) are shown; t-test reference point is gestational week 10+0 – 10+6; ** = p<0.01, *** = p<0.001.

**
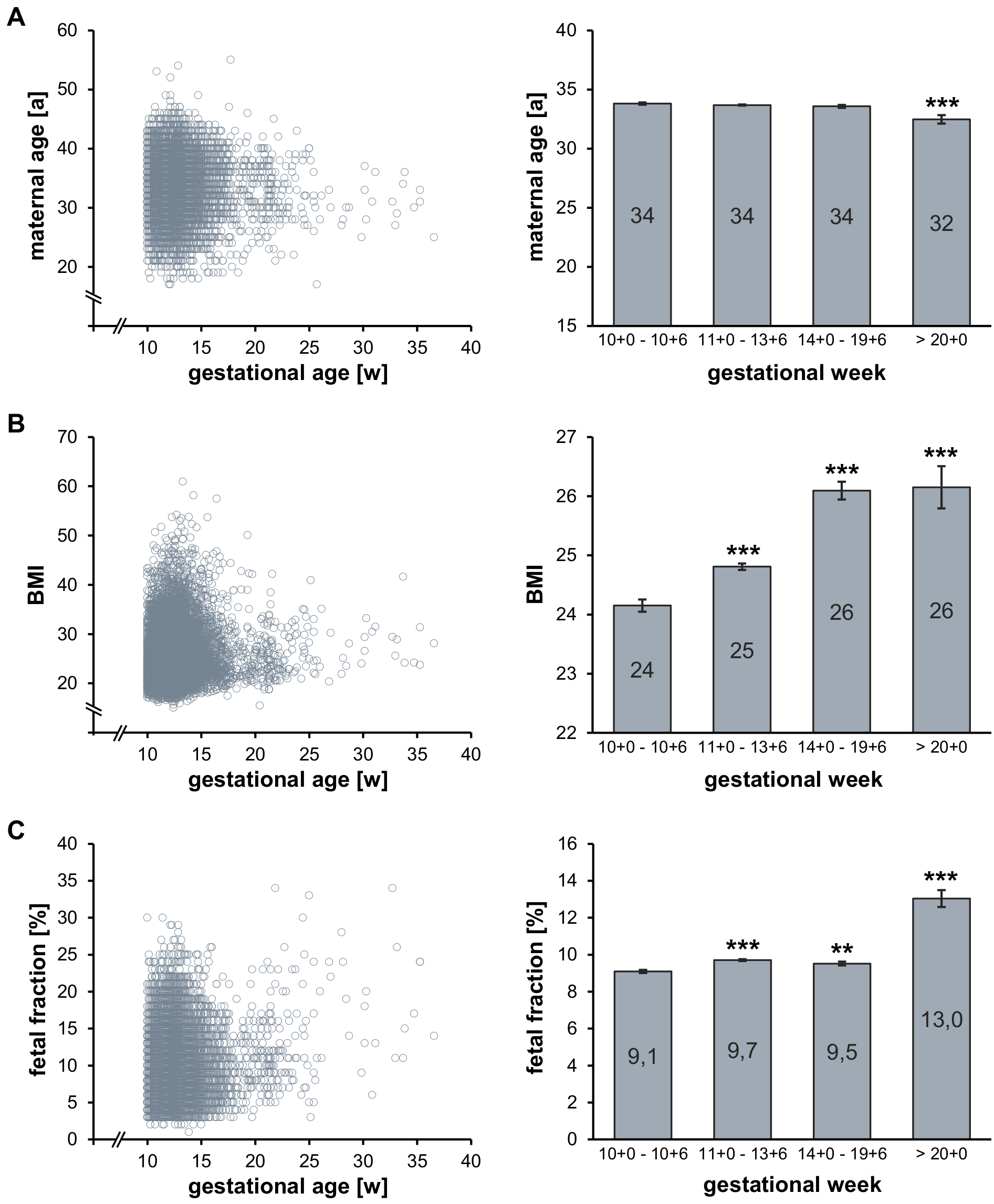
**

**Supplementary Fig. 2**. Relationship between indications for screening and maternal age (A), body mass index (B), and fetal fraction (C). Mean values ± SEM are shown; t-test reference point is adv. maternal age; * = p<0.5, ** = p<0.01, *** = p<0.001.


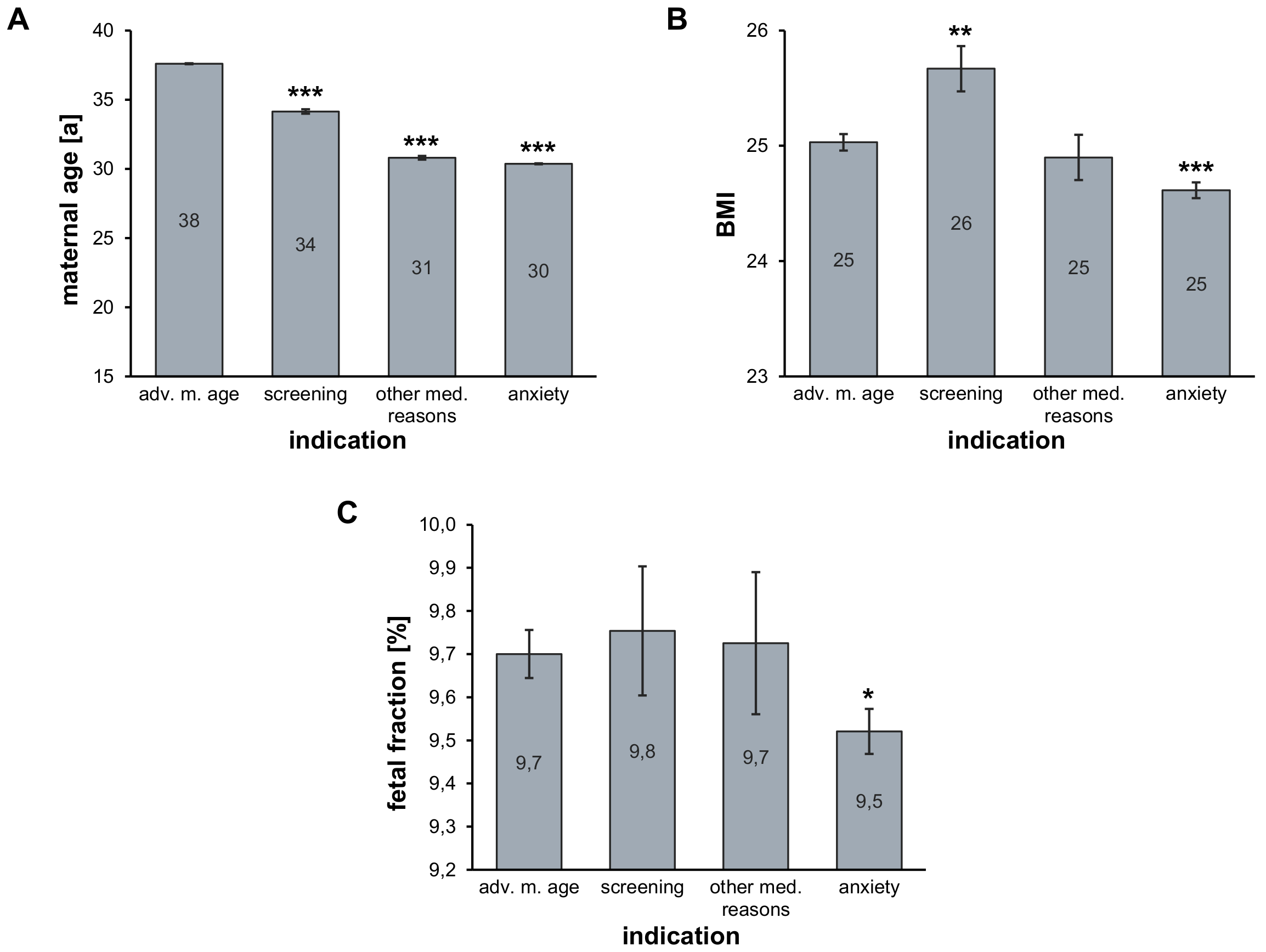


**Supplementary Fig. 3**. Stratification of NIPT results by maternal age (A), body mass index (B), and fetal fraction (C). Mean values ± SEM are shown; t-test reference point is low-risk NIPT result; * = p<0.5, ** = p<0.01, *** = p<0.001.


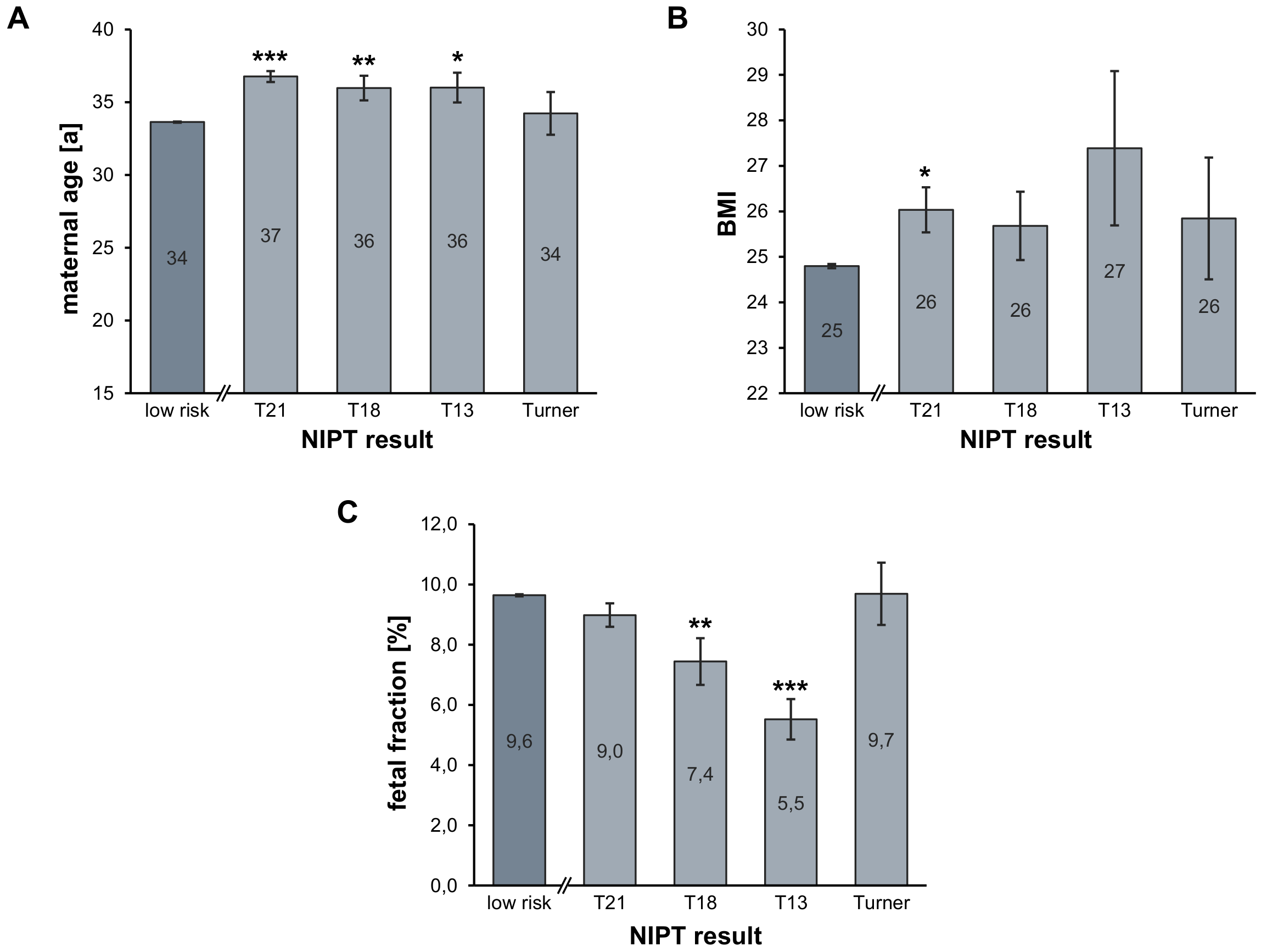


**Supplementary Fig. 4**. Relationship between fetal fraction and patient BMI for low-risk (A) and high-risk (B) NIPT cases. Mean values ± SEM are shown.


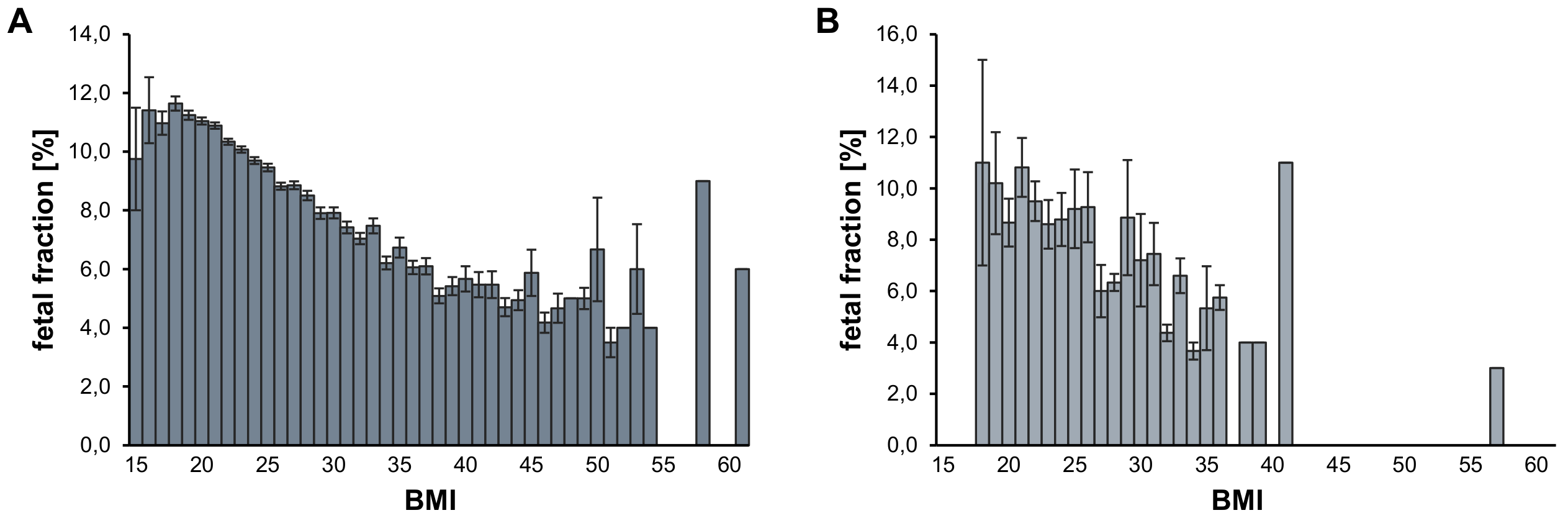

Supplement: Supplementary file 1 — Supplementary file1 (DOCX 56507 kb) [file 404_2020_5856_MOESM1_ESM.docx]
