# Supplementary material for: Analysis of cell-free DNA in a consecutive series of 13,607 routine cases for the detection of fetal chromosomal aneuploidies in a single center in Germany
Source: Arch Gynecol Obstet. 2020 Nov 5;303(6):1407–14. doi: 10.1007/s00404-020-05856-0 (PMC8087552; doi:10.1007/s00404-020-05856-0)
Supplement: Supplementary file 2 — Supplementary file2 (PDF 109 kb) [file 404_2020_5856_MOESM2_ESM.pdf]

SUPPLEMENTARY TABLES

Article Title: Analysis of cell-free DNA in a consecutive series of 13,607 routine cases for the detection of fetal chromosomal aneuploidies in a single center in Germany

Journal Name: Archives of Gynecology and Obstetrics

Author Names: Heike Borth[1], Anna Teubert[2], Ralf Glaubitz[2], Sarah Knippenberg[2], Nargül Kutur[1], Thomas Winkler[1], Bernd Eiben[1*]

Author Affiliations:

[1]amedes Institut für Labormedizin und Klinische Genetik Rhein/Ruhr, Essen, Germany

[2]amedes genetics, Georgstr. 50, D-30159 Hannover, Germany

Corresponding Author:*Bernd Eiben, eiben@eurogen.de

Supplementary Table 1. Clinical follow-up results for low-risk and high-risk cases.

	Outcome results based on clinical follow-up						
	**No aneuploidy detected**	**Trisomy 21**	**Trisomy 18**	**Trisomy 13**	**Trisomy 21 + 13**	**Monosomy X**	**Total**
Invasive diagnostics	30	84	16	8	1	4	143
Amniocentesis	20	61	11	8	1	3	104
CVS	5	16	4	0	0	0	25
Amniocentesis + CVS	0	2	0	0	0	0	2
Unknown*	5	4	1	0	0	1	11
Postnatal cytogenetics	0	1	0	0	0	0	1
Ultrasound	5	5	3	1	0	0	14
New-born physical exam	2,464	1	0	0	0	1	2,466
**Total Cases**	2,499	90	19	9	1	5	2,623

CVS, chorionic villus sampling

*No information about the type of invasive prenatal diagnostic procedure

Supplementary Table 2. Relationship between NIPT results and LLR scores/T-Statistics values.

NIPT Result	True Positives		False Positives	
	**LLR Score**	**T-Statistics Value**	**LLR Score**	**T-Statistics Value**
Trisomy 21	151.8 ± 13.6	15.5 ± 0.7	27.3 ± 23.8	5.2 ± 2.1
Trisomy 18	167.7 ± 43.5	16.2 ± 2.1	109.8 ± 66.9	13.7 ± 5.8
Trisomy 13	125.2 ± 36.0	14.8 ± 2.7	73.5 ± 43.0	10.2 ± 3.7
Trisomy 21+13	N/a	N/a	N/a	N/a
Monosomy X	25.1 ± 8.5	-18.0 ± 2.7	27.4 ± 4.3	-17.4 ± 1.5
All	147.1 ± 12.3	14.2 ± 0.9	54.9 ± 18.4	3.0 ± 2.9

N/a, not available
